# Functional Repurposing of Magnetic Nanoparticle‐Assisted Hyperthermia for Immune Cell Labelling and Tracking

**DOI:** 10.1002/smtd.202502416

**Published:** 2026-06-11

**Authors:** Giulia E. P. Nucci, Chiara Vitale, Alessandro di Girolamo, Niccolò Silvestri, Stefano Persano, Lorenci Gjurgjaj, Cristina Bottino, Roberta Castriconi, Teresa Pellegrino

**Affiliations:** ^1^ Italian Institute of Technology Genoa Italy; ^2^ Department of Experimental Medicine (DIMES) University of Genoa Genoa Italy; ^3^ The Open University Affiliated Research Center Italian Institute of Technology Genoa Italy; ^4^ Clinical and Experimental Immunology Unit, Department of Services IRCCS Istituto Giannina Gaslini Genoa Italy

**Keywords:** cell labelling, immune cells, magnetic hyperthermia, magnetic nanoparticles, magnetic particle imaging

## Abstract

Immune cell therapies show promise in hematologic malignancies and are becoming more effective in solid tumors like lungs, melanoma, and colorectal cancers. Magnetic nanoparticles (MNPs) are mostly used as tracers via magnetic particle imaging(MPI) or magnetic resonance imaging(MRI), and heat mediators to monitor and kill cancer cells via magnetic hyperthermia (MHT). Here, we apply a mild‐MHT protocol (38–41°C/60 min) as a new means to boost MNPs uptake by immune cells under a harmless alternating magnetic field (AMF). Various MNPs formulations, including iron oxide or zinc‐ferrite compositions stabilized by different polymers, were selected for their heating performance and colloidal stability. NK‐92 cell line and NK cells from healthy donors exhibited enhanced MNP internalization upon AMF exposure without compromising viability, receptor expression, and effector functions. MNP‐loaded NK cells retained cytotoxic activity, degranulating upon ligand engagement on glioblastoma and neuroblastoma targets. Similar outcomes were achieved with T cells. Thanks to its simplicity, this MHT‐protocol is suitable for clinical translation, as it can be straightforwardly integrated into existing GMP‐compliant adoptive cell therapy workflows in a hospital cell factory, enabling the preparation of magnetically tagged immune cells ready for tracking in cell therapy infusion.

## Graphical abstract

We propose mild‐MHT protocol for the quick internalization of magnetic nanoparticles (MNPs) by immune cells. The heat induced by MNPs under alternating magnetic field stimulate MNPs diffusion and cell membrane invagination, accelerating the cellular internalization of MNPs.

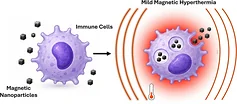

## 1 Main

Immunotherapy is an advanced cancer treatment that boosts the patient's anti‐tumor immune response through strategies such as vaccination, induction of immunogenic cell death, and adoptive cell therapy (ACT) with cytotoxic functions [1]. Natural killer (NK) cells, as part of the Innate Lymphoid Cell family, play a pivotal role in cancer immune surveillance [2]. They recognize and eliminate abnormal or stressed cells by integrating signals derived from a balance of inhibitory and activating surface receptors [3]. Activation is mediated by Natural Cytotoxicity Receptors, NKp46, NKp30, NKp44, as well as other activating receptors including NKG2D, DNAM‐I, 2B4, NKp80, NTB‐A, and low‐affinity IgG receptor III‐A (FcγRIIIa, CD16), which mediates antibody‐dependent cytotoxicity [4, 5, 6]. Upon activation, NK cells release perforin and granzymes from cytolytic granules with consequent surface expression of CD107a (LAMP1), a well‐established marker of degranulation [7, 8]. Inhibitory signals mainly arise from killer cell immunoglobulin‐like receptors (KIRs), binding Human Leukocyte Antigen (HLA) class I molecules (KIR ligands) [9], and NKG2A/CD94, which recognizes non‐classical HLA‐E molecules [10]. Cytokines can further shape the degree of NK cell activity [11].

ACT, using allogeneic NK cells, has shown promising results in haematological malignancies and has yielded encouraging outcomes in some solid tumors such as lungs and melanoma [12, 13]. However, the immune‐suppressive microenvironment often limits immune infiltration and impedes effective therapy delivery [14, 15]. Recent evidence suggests that combining immunotherapies with strategies overcoming immune evasion may enhance therapeutic outcomes [16]. Nanoparticles (NPs) are extensively studied as drug delivery systems due to their capacity for targeted delivery, protection from degradation, enhanced tumor retention, and controlled release via stimuli‐responsive platforms (e.g., pH, magnetic fields) [17, 18, 19, 20, 21]. Moreover, some exhibit intrinsic immunomodulatory properties [22]. Magnetic nanoparticles (MNPs), already used clinically, have garnered increased attention due to their traceability via Magnetic Resonance Imaging (MRI) or Magnetic Particle Imaging (MPI), and their ability to generate heat in magnetic hyperthermia therapy (MHT) under patient‐safe and remotely activated alternating magnetic fields (AMF) without depth‐related signal loss [23, 24, 25, 26]. In clinical trials, MHT has been used as an adjuvant to chemo‐ or radiotherapy for glioblastoma, prostate, and pancreatic cancers, and early studies suggest it may also enhance immunotherapy by promoting immune activation and infiltration via heat‐induced vasodilation [27, 28, 29]. For instance, mild MHT has been shown to boost U87 cell immunogenicity, promoting macrophage phagocytosis and NK cell‐mediated cytotoxicity through modulation of stress‐induced ligands [30].

While most approaches rely on intratumor injection of MNPs, establishing an effective system for their targeted delivery remains a major challenge in nanomedicine. In this context, immune cells, among which NK cells, offer a promising alternative as natural carriers, acting as “Trojan Horses” to enhance MNP delivery to tumors. When labelled with MNPs, NK cells act as both nanoparticle carriers and intrinsic imaging agents, enabling immune cell infiltration tracking and quantification via MPI [31, 32]. This multifunctionality offers real‐time monitoring of cell dynamics and tumor engagement, providing valuable insights to optimize immunotherapy strategies.

To promote MNPs internalization by immune cells, co‐incubation, chemical binding, and application of static magnetic fields have been explored, so far [4, 5, 6]. For instance, Sanz‐Ortega et al. labelled NK cells with iron oxide NPs via co‐incubation at 37°C for 24 h, preserving viability and effector functions in both human and murine NK cells [33]. Similarly, Li et al. used FDA‐approved Ferumoxytol, achieving an iron uptake of 4 pg/cell and in vivo MRI detection [34]. Sim et al. further improved this approach by combining Ferumoxytol with hyaluronic acid and protamine, enabling effective labelling without toxicity and with MRI signal visibility [35]. Other methods include polydopamine‐coated NPs, used by Wu et al., though these co‐incubation methods lacked specificity and required prolonged (minimum12 h) incubation time [36]. Burga et al. proposed a “biohybrid” strategy to anchor iron oxide MNPs to NK cell membranes while maintaining cell integrity [37]. Jang et al. used a magnetic field gradient to force MNPs into cells, but this technique, based on magnetic attraction rather than MHT, is less adaptable to non‐adherent immune cells in suspension [38]. Most existing protocols use NK cell lines, which differ substantially from primary NK cells from healthy donors (HDs) or cancer patients [42]. Moreover, most of these studies required several hours of incubation to achieve optimal uptake at the picogram scale. Few studies have addressed intracellular MNP uptake in healthy donor (HD)‐NK cells, which are smaller and less prone to nanoparticle internalization, posing challenges for protocol optimization [38].

**SCHEME 1:**
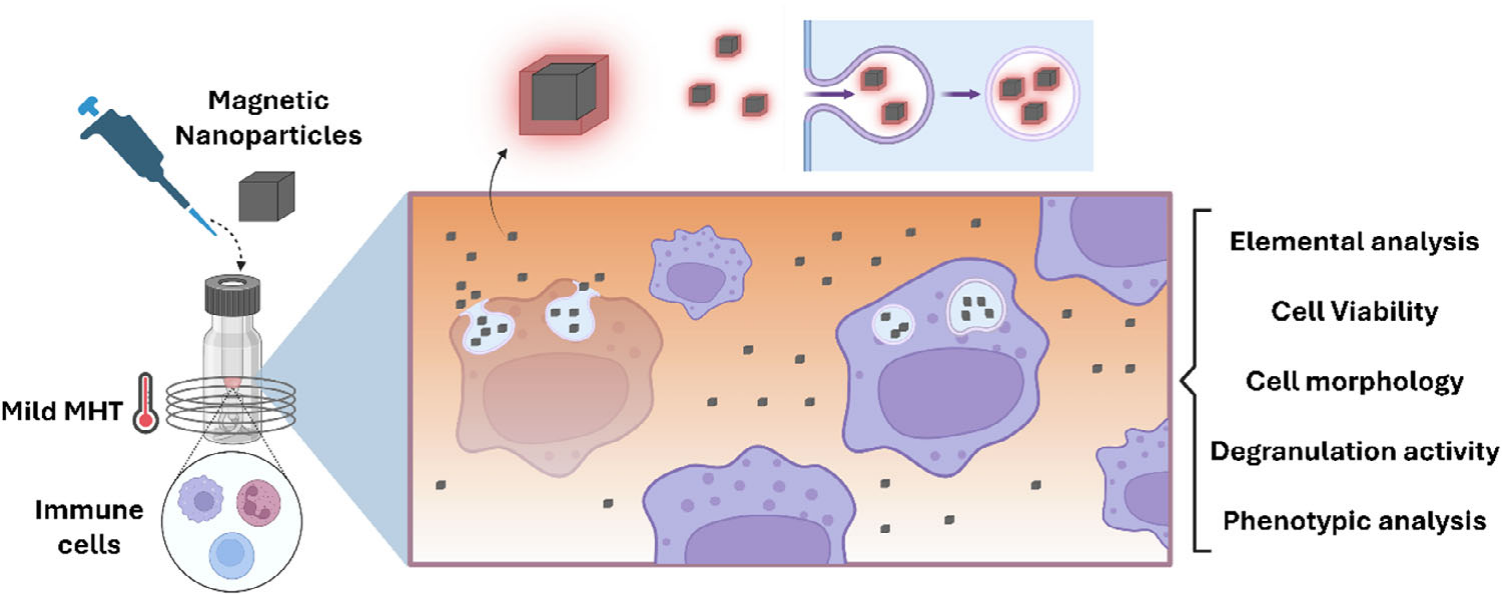
Schematic drawing of the overall mild‐MHT‐based protocol for MNPs internalization that we have developed here. Immune cells (NK92, or HD‐NK, or T‐cells) and MNPs are mixed in a septum‐closed glass vial and subjected to two cycles of 30 min of mild‐MHT. The soft heat (in the range 38–41°C) reached under AMF exposure is used to promote cell uptake and nanoparticles’ diffusion. Upon mild‐MHT treatment, some tests are applied to assess the uptake and status of the immune cells.

Here, we propose a novel and quick approach to enhance MNPs integration into immune cells by applying mild‐MHT, exposing cells to a moderate temperature range (38–41 °C) under an AMF for only 1 h, making the process faster and more efficient than any other method so far used. The mild heat promotes cell membrane endocytosis, facilitating MNP diffusion without inducing toxicity or compromising cell integrity (Scheme 1). The protocol, initially optimized for the NK‐92 cell line (the latter line also used in clinical trials), is successfully extended to HD‐NK cells and T cell lines using different MNP formulations, demonstrating an efficient and rapid uptake, its broad applicability across different immune cell types, and magnetic materials.

### 1.1 Identifying Optimal Parameters for Mild‐MHT Applications

**FIGURE 1:**
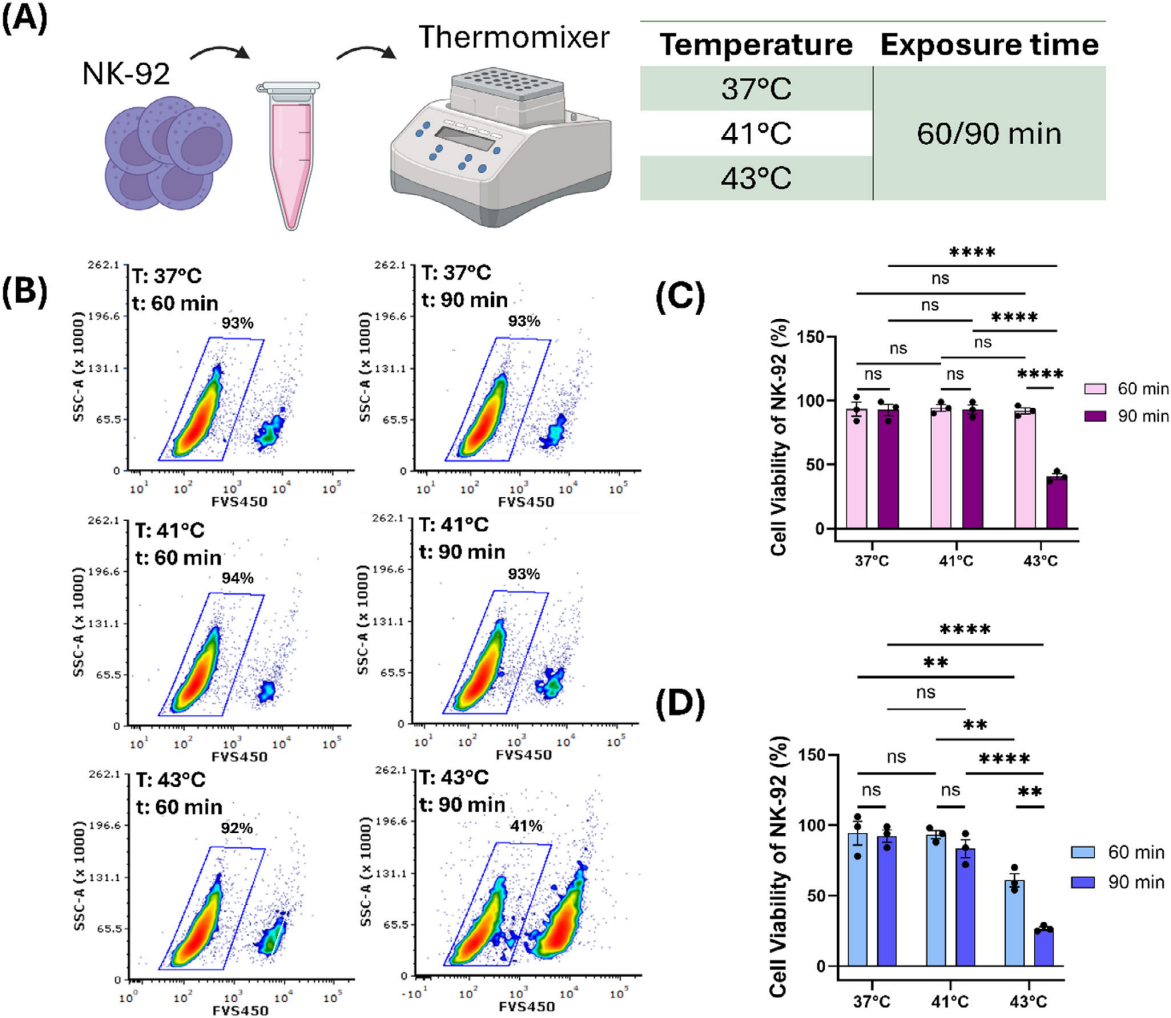
Thermo‐tolerance assay of NK‐92 cells. (A) Schematic representation of the heat experiment to evaluate the thermo tolerance of solely NK cells when exposed to specific temperature values and duration times. NK92 cells (only) were incubated in the thermomixer used as a heating source set at different temperature values (37°C, 41°C, or 43°C) for different times (60 or 90 min) before measuring cell viability 24 h later. (B) Representative cytofluorimetric analysis of the viability of NK‐92 cells collected after thermomixer treatment and upon further incubation at 37°C for 24 h. The square boxes highlight live cells (FV450‐negative cells in the blue frame). (C) Histogram graph of the percentage of viable NK‐92 cells analysed (mean ± SEM with N = 3) at C) 24 h or (D) 48 h post‐exposure to the indicated thermomixer temperature increase. Statistical significance was analysed by the two‐way ANOVA test. ^**^ *P* < 0.01, ^****^ *P* < 0.0001, ns = not significant, SEM = standard error of the mean.

To define optimal temperature conditions that maximize MNP uptake without impairing immune cell functionality, we first conducted a preliminary thermal tolerance study on solely NK‐92 cells and using a thermomixer as a heat source. One million cells were exposed to either 41°C or 43°C for 60 or 90 min (Figure 1A). Viability was analysed 24 h post‐treatment by flow cytometry (Figure 1B,C). Cells maintained viability higher than 90% following exposure to 41°C (60 or 90 min) and 43°C (60 min), compared to untreated controls at 37°C. However, temperature treatment at 43°C for 90 min significantly reduced viability (∼43%). After 48 h, 41°C‐treated cells maintained good viability, while 43°C exposure further reduced survival (64% at 60 min; 29% at 90 min) (Figure 1D). Optical microscopy supported these findings: 41°C‐treated cells remained proliferative, while those at 43°C for 90 min showed high mortality and disrupted clustering (Figure S1). Overall, these data identified 41°C for 60 min as the optimal temperature and time duration to preserve NK cell viability and physiological functionality and to translate to MHT for a mild heat treatment condition.

### 1.2 Selection of the Best‐Performing MNPs

Next, we replaced the thermomixer with AMF, thus adding the MNPs to the cells to implement an MHT‐based heat protocol. MNPs heating ability is influenced by MNPs parameters, including size, shape, surface coating, composition, and by AMF parameters. We selected cubic‐shaped MNPs of either iron oxide (Fe_3_O_4_, referred to as IONCs) or zinc ferrite (Fe_2.8_Zn_0.2_O_3_, referred to as Zn‐Fe) with a cube edge size of 15–18 nm. This subset of MNPs was chosen because they achieve optimal heating performances, measured as specific absorption rate (SAR) values, and show superparamagnetic behavior at room temperature, so they are easily to handle in absece of any magnetic field [39, 40, 41].

**FIGURE 2:**
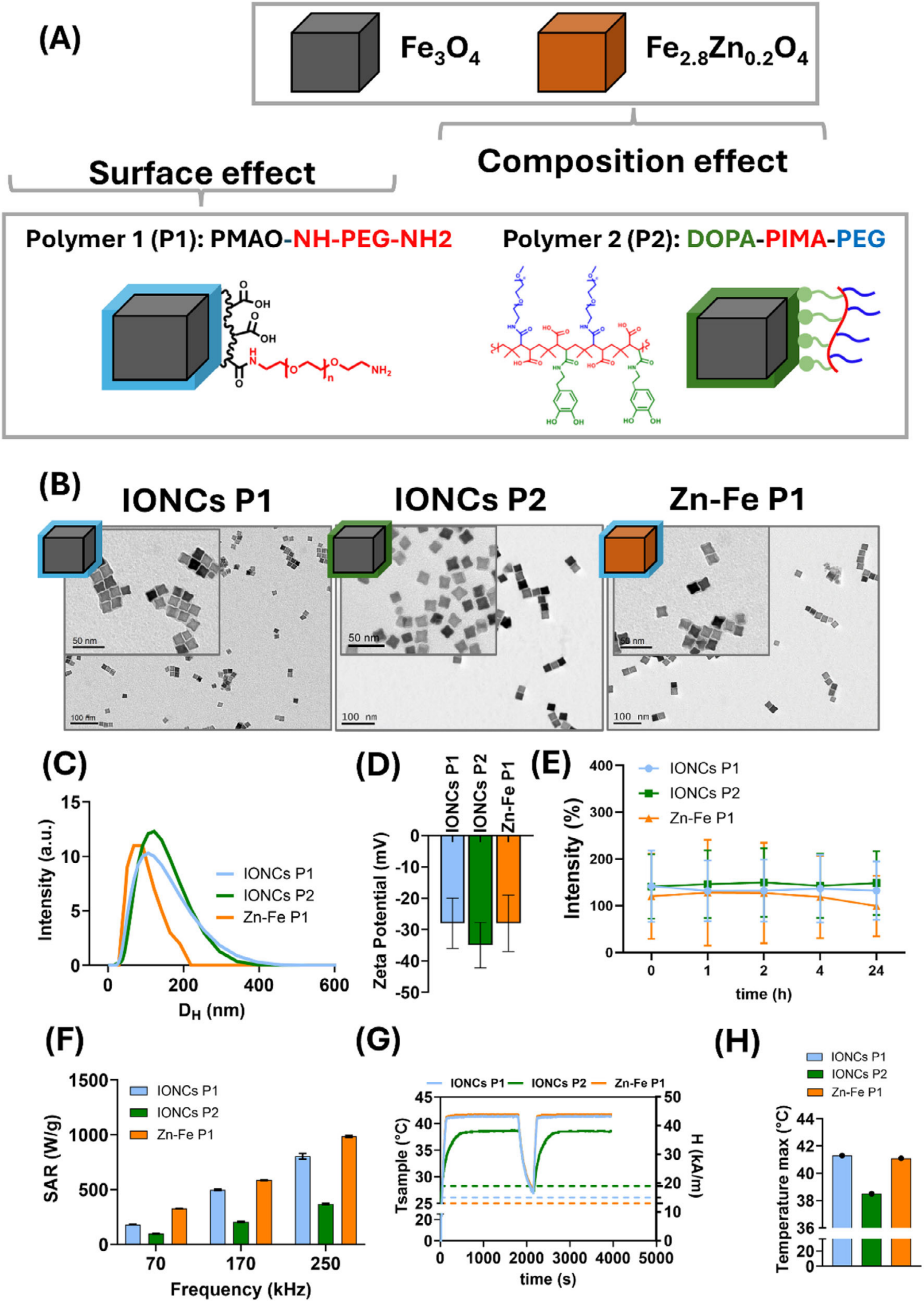
Characterization of MNPs selected for mild‐MHT. (A) Schematic cartoons of composition and polymer coatings chosen for the MHT experiments. (B) TEM images of the water‐transferred MNPs, along with their (C) relative hydrodynamic DLS size and (D) surface charge in water, are shown. (E) The stability in complete cell culture medium was measured through DLS profile weighted by intensity. (F) SAR values of the selected MNPs at different frequencies. (G) Temperature vs. time profiles during the two MHT exposure cycles for the selected MNPs; the maximum value of H reached by the three MNPs samples is different (H). Histogram showing the temperatures reached during the treatment by the three different MNPs.

Such MNPs were synthesized in an apolar solvent and subsequently transferred from chloroform into water to ensure dispersion in cell culture media (Figure 2A) [40, 42]. For the first selected coating, here referred as polymer 1 (P1), surface functionalization involved a first coating step with an amphiphilic poly(maleic anhydride‐alt‐1‐octadecene) (PMAO) polymer via hydrophobic interdigitation of polymer alkilyc chains and alkilyc surfactant molecules at the MNPs surface followed by a second conjugation step of diamine–polyethylene glycol molecules (NH_2_–PEG–NH_2_, MW 2000) through EDC chemistry to the PC coated MNPs, involving the carboxyl groups of the hydrolysed PMAO shell and the amino groups of NH_2_–PEG–NH_2_ (Figure 2A).

As an alternative, we have selected a multi‐anchoring ligand polymer employing polyisopropyl maleic anhydride polymer pre‐functionalized with dopamine units and 2000 MW amine‐PEG methoxy chains, here referred to as polymer 2 (P2) (Figure 2B) [43]. Both coatings produced stable, individually dispersed nanocubes in water, as confirmed by a single monolayer of MNPs under TEM (Figure 2B), with monomodal hydrodynamic profiles (PDI ≤ 0.20) and Dynamic Light Scattering peaks below 200 nm (Figure 2C and Table S1). Zeta potential measurements confirmed that all MNPs carried a negative surface charge: −28 ± 7 mV (IONCs P1), −53 ± 9 mV (IONCs P2), and −39 mV (Zn‐Fe P1) (Figure 2D). Furthermore, water‐transferred MNPs remained stable in complete culture medium at 37°C for 24 h, showing no aggregation through electrostatic repulsions of the carboxyl groups of the polyanhydrides and the steric hindrance of the PEG chains (Figure 2E) [43, 44].The colloidal stability of MNPs in the cell media is fundamental to promote immune cell MNPs interactions without compromising the uptake, toxicity, and MNP heat effect. We next evaluated MHT‐heating capabilities via AC magnetometry across 70–250 kHz at 24 kA/m (Figure 2F). IONCs P1 reached SAR values of ∼200, 500, and 800 W/g at 70, 110, and 250 kHz, respectively, while IONCs P2 exhibited slightly lower SARs (∼100, 200, 500 W/g). Zn‐Fe P1 showed the highest SAR values (∼300 W/g at 110 kHz, 900 W/g at 250 kHz) and reached 41°C at lower field intensities (∼12 kA/m at 3 g_Fe_/L), indicating superior heating efficiency than commercially available materials or other synthesized materials [41]. To replicate mild‐MHT conditions, meaning 41°C, we have first investigated the correlation between MNPs concentration and temperature profile: at fixed 182 kHz frequency, all samples (3 g_Fe_/L) were subjected to two 30 min MHT cycles with field intensity auto‐adjusted by the device at the steady values in the range between 17–24 kA/m such that the optimal temperature of 41°C was maintained for at least 1 h (Figure 2G and Figure S2 for a zoom of the H profiles overtime). IONCs P1 and Zn‐Fe P1 rapidly reached and sustained 41°C, while IONCs P2 only reached 38°C, likely due to its different polymer coating (Figure 2G,H). Despite this, we purposely selected both IONCs P1 and P2 to test MNPs uptake under mild‐MHT, reaching 38°C and 41°C, having both samples comparable stability in culture medium (Figure 2E).

### 1.3 Heat From MNPs in Mild‐MHT Boosts Intracellular Uptake

**FIGURE 3:**
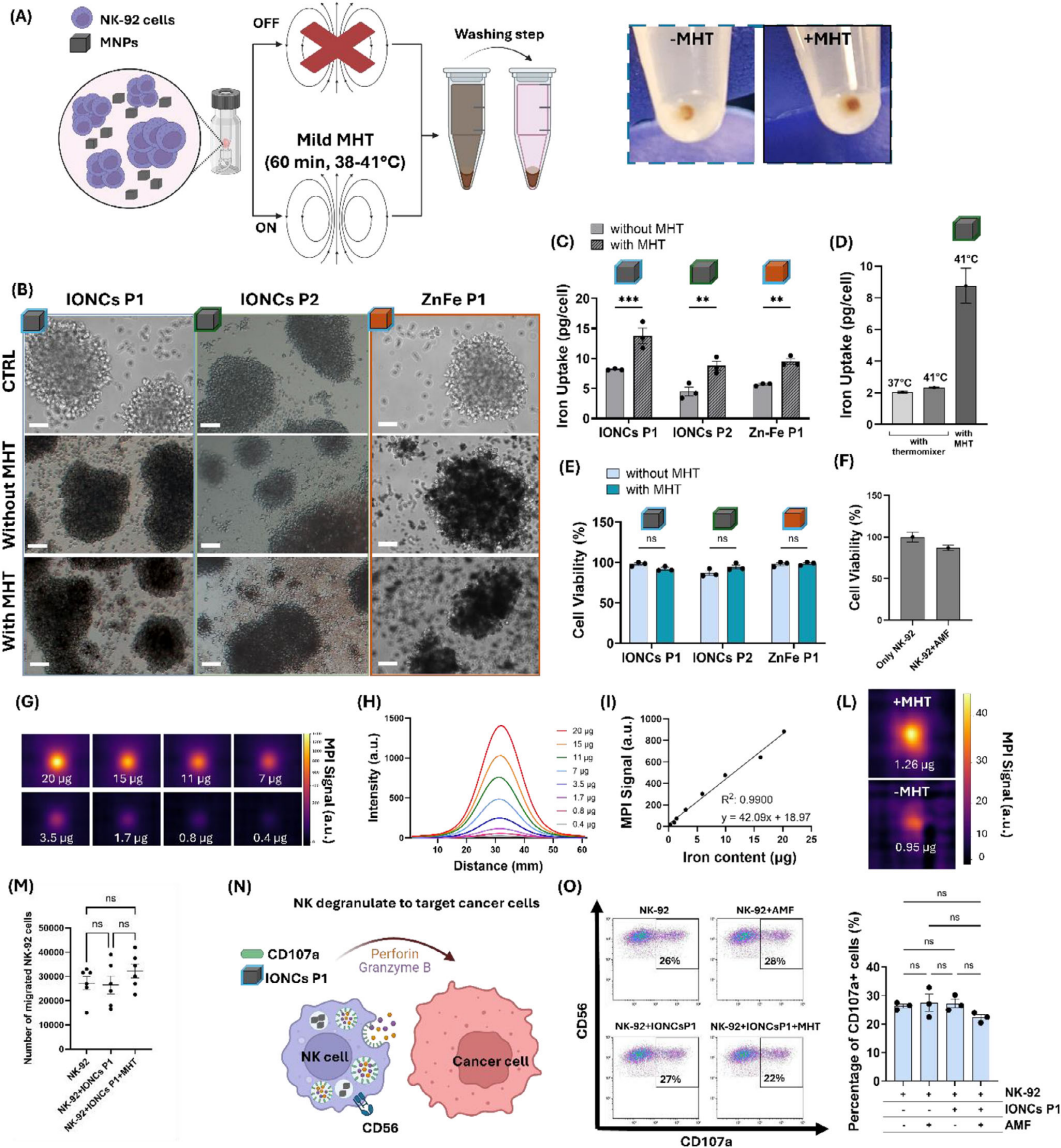
Iron uptake, viability, and functional analysis on MNPs‐doped NK‐92 cells. (A) Representative sketch of the uptake protocol with or without the mild‐MHT treatment. Cells were washed twice to remove all the nanoparticles that were not associated with cells, and the pellet, visually inspected, showed a darker color for cells undergoing MHT cycles. (B) Optical images of NK‐92 were collected 24 h after exposure to the MHT protocol for all the MNPs used. The dark spots associated with the cells appear as a distinct but qualitative sign of the association of magnetic materials with the cells when treated with MNPs with or without exposure to mild‐MHT. (C) The pellet was then processed to quantitatively evaluate the Fe content through ICP‐OES elemental analysis. Elemental Iron expressed in Fe uptake per cell (pg/cell) for the three different samples. (D) Elemental analysis expressed as Fe uptake per cell (pg/cell) for the NK‐92 cells incubated with IONCs P2 at 37°C or at 41°C using a thermomixer as an alternative heat source, compared with mild MHT‐treated samples reaching 41°C. (E) Cell viability by FVS450 assay, plotted as mean ± SEM of the percentage of alive cells per sample, indicates no cytotoxicity even in the presence of AMF and MNPs. (F) Cell viability of the control group, NK‐92 cells at 37°C alone, and NK‐92 in the presence of AMF (to note that under AMF and NK‐92 cells only, there is no temperature increase under AMF, Figure S4) (G) Example of MPI projection images of IONCs P2 used to build the calibration. (H) Line profiles along the same diameter of the region of interest (ROI) show differences in signal intensity across all the samples analysed. (I) Calibration curve correlating the iron content with the corresponding MPI signal. (L) MPI images of NK‐92 with IONCs P2 and exposed to MHT or NK‐92 cells with IONCs P2 and incubated at 37°C. (M) Migratory capacity of NK‐92 cells after mild‐MHT in different conditions: NK‐92 cells alone, NK‐92 in the presence of IONCs P1, and NK‐92 with IONCs P1 and exposed to 2 cycles of MHT (30 min each). (N) Scheme showing the degranulation activity of NK cells in the presence of cancer cells. (O) NK‐92 cells, treated as indicated, were cocultured with U87 target cells (1:1 effector: target ratio), and degranulation capability was analysed by flow cytometry (percentages of CD107a positive cells. On the left panel, the squared plot shows the percentages of CD107a+ CD56+ NK cells. On the Right panel, the mean percentage of CD107a+ cells of three independent experiments for sample IONCs P1. Statistical significance was analysed by a two‐way ANOVA test. ns = not significant. Scale bar in optical images: 100 µm.

We then proceeded with setting mild‐MHT experiments in NK‐92 cells. UV‐sterilized MNPs were added to one million cells to achieve a final concentration of 3 g Fe/L (Figure 3A). Cell's samples were exposed to AMF (f = 182 kHz, H = 17–24 kA/m) for 60 min, and, after the treatment, cells were washed to remove excess MNPs. It is worth noting that the mild‐MHT protocol has been tested at H·*f* product that remains within the limits considered acceptable for small‐volume exposures but exceeding the Brezovich limit (H*f ≤ 4.85*10^8^ A*m ^−1^*s ^−1^). However, given the ex vivo nature of the protocol and the extremely small sample volume (150 µL), any heating contribution from tissue eddy currents, as observed in the case of in vivo application in a patient, is not relevant for our protocol. Moreover, contrary to in vivo protocols, our protocol under AMF takes only a short time (1 h), a time span that again is not enough to compromise the immune cells, as shown in Figure 3F and Figure S4 [45, 46, 47]. At a first qualitative visual inspection, pellets from MHT‐treated cells appeared darker brown than those incubated with MNPs at 37°C, suggesting greater MNPs association (Figure 3A). To quantify MNP uptake, iron was determined through elemental analysis by Inductively Coupled Plasma Optical Emission Spectroscopy (ICP‐OES) after acidic digestion of the pellets. MHT significantly enhanced MNPs uptake compared to non‐exposed cells (Figure 3C). Specifically, iron content in NK‐92 cells treated with IONCs P1 and IONCs P2 under MHT reached ∼13 pg and ∼9 pg Fe/cell, respectively, a 1.6 to 1.8 fold increase over cells incubated with MNPs at 37°C without MHT (8 pg for P1, 5 pg for P2). A similar trend was observed with Zn‐Fe P1 MNPs (Figure 3C). Importantly, despite the potential reduction in heating efficiency typically associated with MNPs intracellular clustering, in our conditions, MNPs internalization did not significantly impact the experimental field intensity conditions on cells (Figure S3A) [48, 49]. In principle, as MNPs become internalized, one would expect a change in the applied field intensity to maintain the target temperature of 41°C. However, in our protocol, the internalized nanoparticle fraction in cells represents only ∼4% of the total MNP mass administered to the cells (18 vs. 450 µg of iron administered to the cells), which explains why no significant adjustments in the magnetic field amplitude were required during the 1‐h AMF protocol to maintain the target temperature for the cells sample in the presence of IONCs (Figure S3, ESI). Moreover, after 24 h, all conditions showed good viability (>80%) with no significant differences between control and treated cells, confirming the biocompatibility of both MNPs and the mild‐MHT protocol (Figure 3E). Importantly, the AMF itself was non‐toxic, as cells exposed to AMF (at 182 kHz, 17 kA/m, 60 min) exhibited 90% viability comparable to the 100% viability of cells maintained at 37°C (Figure 3F and S4). Optical microscopy confirmed intact NK92 cell cluster formation across all samples, with no signs of morphological impairment (Figure 3B), supporting the safety and effectiveness of the mild‐MHT protocol and selected IONCs on the cells.

Notably, iron quantification by elemental analysis by ICP revealed significantly higher iron levels in NK cells exposed to 41 °C via MHT (8.7 pg/cell) compared to those heated at the same temperature (41°C) but with a thermomixer (2.3 pg/cell), as shown in Figure 3D. This nearly 4 fold difference confirms that the observed enhancement is not merely due to a thermal effect but is specifically driven by the synergistic action of the MNPs under AMF, suggesting an enhanced MNPs diffusion and transient cellular membrane changes occurring under AMF and MNPs presence. This interpretation is consistent with the presence of local nanoscale heating effects that are not captured by macroscopic temperature measurements. However, the prediction of local temperature at the nanoparticle surface is not straightforward. Continuum approaches (e.g., Baffou et al.) [50] based on diffusive heat transport and bulk thermal conductivities, predict negligible single particle heating but may not be valid at the nanoscale [50]. Indeed, interfacial thermal resistance and non‐diffusive transport in the organic shell (as suggested by Riedinger et al.) at the nanoparticle surface can lead to significantly higher local temperatures than those estimated by classical models [51]. This latter local effect may indirectly justify the difference in MNPs uptake by immune cells observed when using a thermomixer set at 41°C versus an AMF experiment at the same nominal temperature of 41°C as found by us (Figure 3D).

To validate traceability and iron quantification in MNP‐loaded NK cells, we performed preliminary MPI analysis using IONCs P2, which showed the lowest uptake levels. MPI is a non‐invasive, real‐time imaging modality that detects signals exclusively from MNPs, without background noise or tissue attenuation, enabling highly sensitive quantification [52, 53].

We first established a linear calibration curve correlating MPI signal intensity with iron concentration of IONCs P2 in the 0.4–20 µg range (Figure 3G–H and Figure S5). The MPI signal showed a very good linear correlation with iron content (R^2^ = 0.99) (Figure 3I), which we used to quantify iron in NK cell pellets with or without mild‐MHT exposure (Figure 3L). After removing unbound nanoparticles, the total cellular iron content was measured and quantified by MPI signal and it was found 0.95 µg of iron in cells maintained at 37°C and it increased to 1.26 µg following mild‐MHT. These values were consistent with ICP values of iron, which were 0.9 and 2 µg, respectively.

Furthermore, the evaluation of iron content 24 h after the MHT protocol showed that the amount of iron associated with the cell pellet remained stable, with no measurable iron leakage (0 h MHT: 1.01 µg; 24 h MHT: 1.14 µg) (Figure S6). This highlights the potential of such labelled cells for translation into more complex biological environments.

**FIGURE 4:**
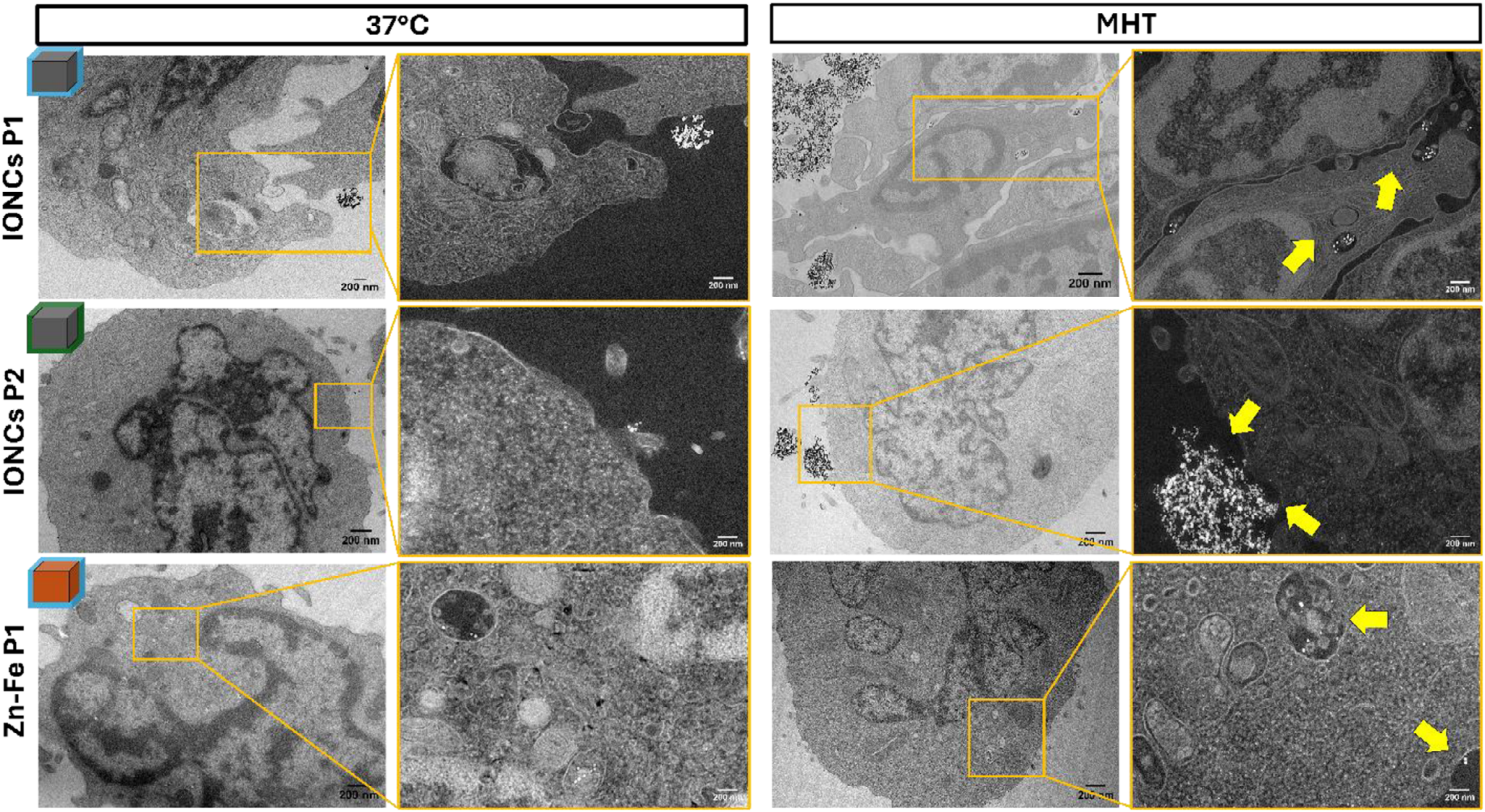
Comparative TEM structural analysis of NK‐92 treated with different nanocubes with mild‐MHT (41°C) or at 37°C. For each condition and MNPs type, we have included a low magnification cell image (first and third columns) and a high magnification digitally inverted image (second and forth columns) to enhance nanoparticle visibility on zoomed cell areas. Scale bars are indicated in each image. At high magnification and especially on digitally inverted images (yellow frames), nanocubes appear as white bright spots (yellow arrows), indicating the location of the nanoparticles both on the cell membrane and in intracellular vesicles.

Moreover, compared with recent labelling studies of NK92 cells, in which standard incubation of cells with MNPs at 37°C for 20 h yielded an uptake of 3.17 pg of iron per cell, our mild‐MHT method achieved ca.13 pg/cell in just 1 h, a 4 fold increase in a much shorter (1/20) fraction of time [54]. Furthermore, to address whether endocytosis/ transient membrane permeabilization could lead to delayed functional impairment, we also evaluated the migratory capacity of NK‐92 cells,18 h post‐treatment. A chemotaxis assay was performed using U87 glioblastoma‐conditioned medium as a chemoattractant in the lower chamber of a transwell chamber, while NK92 cells were placed on top of the transwell chamber on the porous membrane. As shown in Figure 3M, MHT‐treated cells exhibited a migratory profile similar to that of untreated controls, confirming that the cells fully recovered their biological fitness, and they maintained their ability to respond to tumor‐derived stimuli. Next, we assessed the impact of IONCs' internalization on NK‐92 physiological activation. Experiments were conducted with IONCs P1, which showed the highest MNPs uptake. Specifically, we analyzed the degranulation of the NK‐92 cell line cultured in the presence of the U87 glioblastoma cell line. Degranulation of secretory lysosome contents, including perforin and granzyme, is a critical event leading to tumor cell death. This process is primarily triggered by NK‐activating receptors recognizing their specific ligands upon cell‐tumor contact. Importantly, as shown in Figure 3N,O, NK‐92, NK‐92+IONCs, and NK‐92+IONCs+MHT cells exhibited comparable degranulation levels (26%, 27%, and 22%, respectively), indicating that, even in the presence of IONCs uptake or IONCs and AMF exposure together, the NK‐92 cell line fully retains its cytolytic function. These findings were supported by ICP results and further corroborated by TEM imaging, which showed the distinct cubic‐shaped nanoparticles localization as dark (Figure 4, first and third columns) or bright spots in inverted images (second and forth columns). In all conditions, nanocubes were observed on the cell membrane and within endosomal vesicles, with higher visibility at 41°C than at 37°C, suggesting that mild‐MHT enhance MNP uptake/ cell membrane MNPs association.

### 1.4 Consolidating Mild‐MHT Protocol Using HD‐NK and Other Immune Cells

**FIGURE 5:**
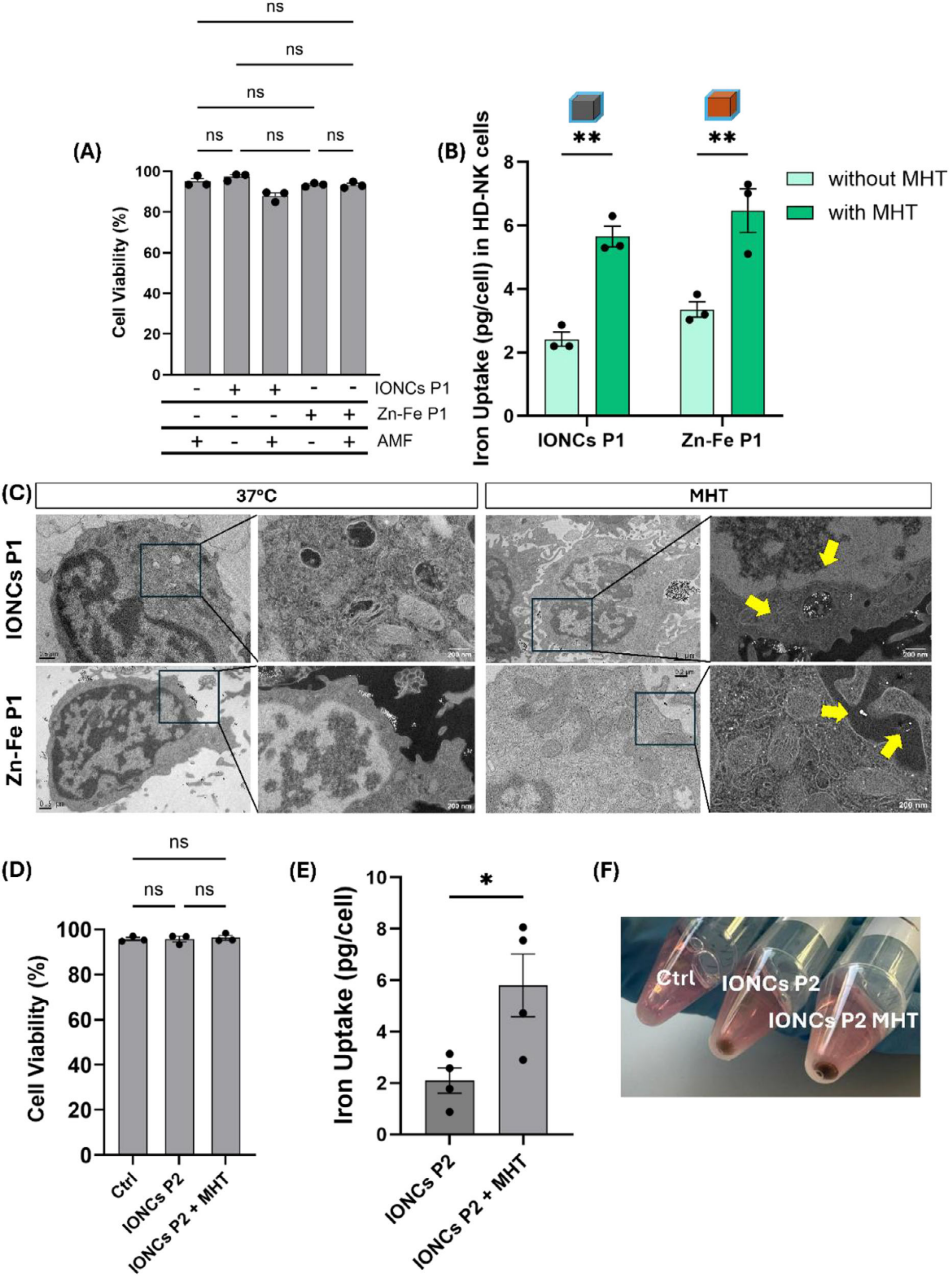
Mild‐MHT uptake protocol extended to primary HD‐NK cells extracted from a healthy donor and Jurkat T cells. (A) Cell viability by FVS450 assay using IONCs P1 nanoparticles, reported as the mean ± SEM of the percentage of alive HD NK cells. (B) Iron uptake measured through IC‐OES analysis and expressed in pg Fe/cell, indicating a significant 3 fold increase when using the mild‐MHT protocol. (C) TEM images of HD NK cells exposed to magnetic nanocubes with or without mild‐MHT. Yellow arrows show the location of the nanoparticles on the cell membrane or intracellular vesicles. (D) mean ± SEM of the percentage of alive T cells per sample. (E) Iron elemental results on T cells expressed in Fe uptake per cell (pg/cell). (F) The different colors of the T cell pellet are shown. Statistical significance was analysed by a two‐way ANOVA test. ns = not significant, ^**^ *P* < 0.01.

Having assessed mild MHT as a safe and effective method to enhance MNP internalization in NK‐92 cells, we validated the approach using primary NK cells purified from blood of human donor and expanded in vitro to generate activated polyclonal populations. For these trials, we focused on the best‐performing MNPs, i.e., IONCs P1 and Zn‐Fe P1 NPs, applying the same protocol conditions previously reported. Also in this case, the mild‐MHT procedure showed a safety profile in terms of viability, as evidenced by the high percentages of viable HD‐NK cells (∼90%) (Figure 5A).

Despite being smaller than NK‐92 cells, HD‐NK cells showed significantly enhanced MNP uptake under MHT, reaching 5.7 pg Fe/cell for IONCs P1 and 6.4 pg Fe/cell for Zn‐Fe P1, as measured by ICP, approximately a threefold increase over control HD‐NK cells kept at 37 °C without AMF exposure (2.3 pg and 3.3 pg Fe/cell, respectively; Figure 5B). TEM imaging corroborated these findings: IONCs were visible on the plasma membrane and within membrane invaginations following MHT, while Zn‐Fe P1 NPs were primarily localized at the cell surface in both treated and control conditions (Figure 5C). Importantly, no morphological indicators of cellular stress or damage, including cell membrane alteration, were observed in HD‐NK cells with or without MHT, further confirming the non‐toxic nature of the treatment and MNPs formulations (Figure 5C). The integrity of the plasma membrane aligns with endocytosis as the main mechanism responsible for MNP internalization. In fact, the receptor‐independent endocytosis mainly occurs through protrusions of the intact plasma membrane.

To evaluate the persistence of the MNPs association, iron content was measured 24 h post‐treatment by ICP‐OES (Figure S5). The results showed no decrease in intracellular iron levels (5.7 pg Fe/cell) at 24 h, indicating stable retention of internalized nanocubes over time (Figure S5).

**FIGURE 6:**
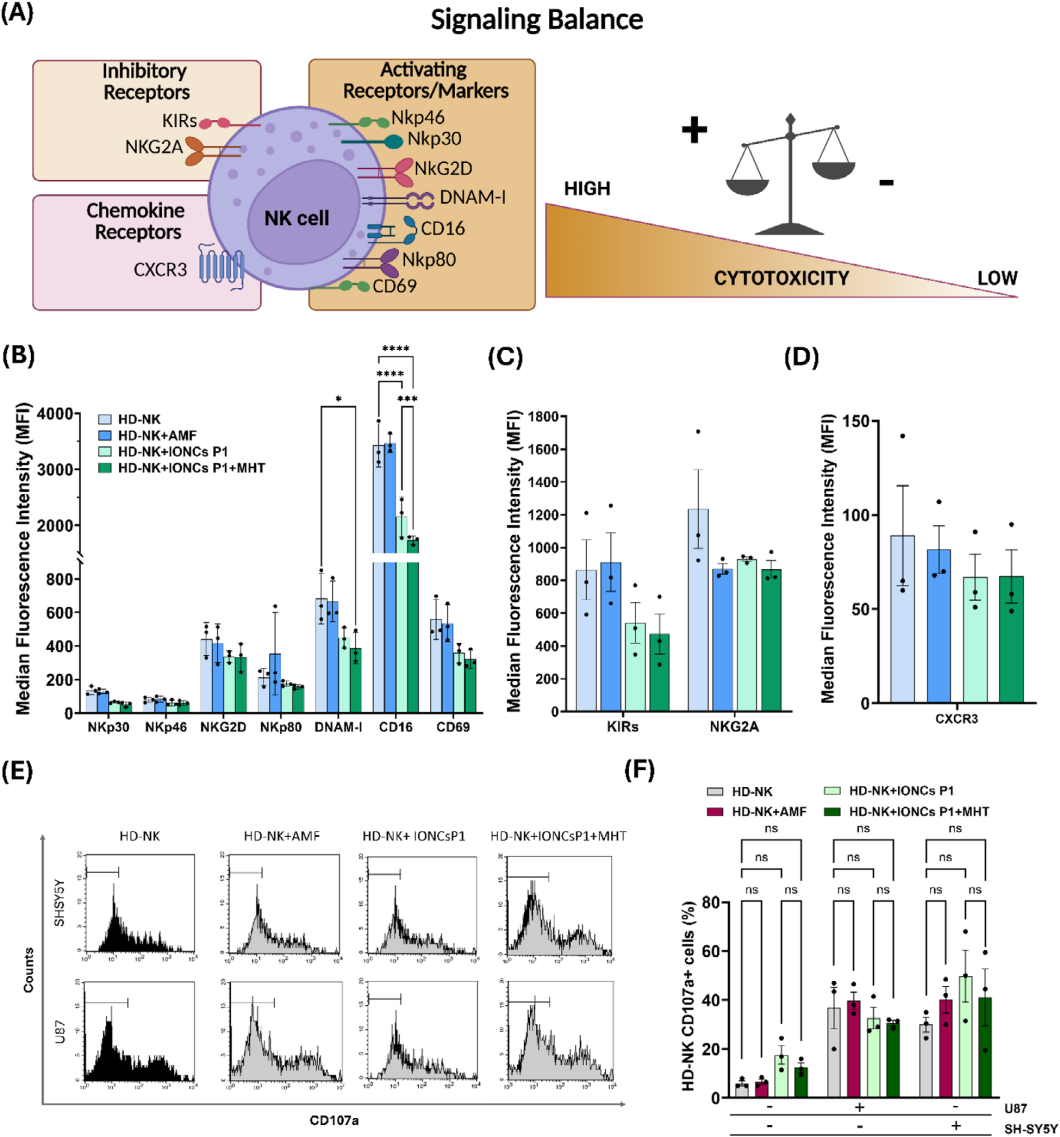
Phenotypic and functional analysis of HD‐NK after incubation with IONCs P1 and MHT. (A) Scheme of the signalling balance regulating NK cell interactions with potential targets such as cancer cells. The main inhibitory or activating receptors are highlighted. (B‐C‐D) Phenotypic results from three independent experiments assessing the expression in HD‐NK cells of the relevant surface markers. The analysis was conducted by testing different conditions: untreated cells (control), HD‐NK cells + AMF, HD‐NK cells + IONCs P1, and HD‐NK cells + IONCs P1 + MHT. (E) Representative flow cytometry analysis of the degranulation capability of HD‐NK cells, untreated or treated with IONCs P1 ± 2 cycles of MHT. Targets were represented by neuroblastoma (SH‐SY5Y) or glioblastoma (U87) cell lines. (F) Degranulation results from three independent experiments. All the histograms show the mean ± SEM from three independent experiments, each performed in triplicate. Statistical significance was analysed by a two‐way ANOVA test. ^*^ *p* < 0.05, ^**^ *p* < 0.01, and ^****^ *p* < 0.0001. Only statistically significant differences are highlighted in the graph.

To further investigate whether the association of nanocubes affects NK cell function, we first evaluated the expression of key activating and inhibitory receptors on HD‐NK cells, ensuring that MNPs adhesion and internalization did not alter their phenotype. As NK cell function relies on a balance of inhibitory and activating signals, including the activation marker CD69 (Figure 6A), it is critical to preserve the receptor profile.

Moreover, we also assessed the level of expression of the activation marker CD69, as an indicator of cellular activation, and the chemokine receptor CXCR3, which is a relevant chemokine receptor involved in the migration of NK cells from the periphery to sites of inflammation. This is particularly relevant if NK cells are to be used as “Trojan horses” for MNP delivery to tumors, where migratory capacity and surface marker integrity are essential. To this aim, HD‐NK cells were subjected to different conditions: untreated (37°C) (HD‐NK), exposed to AMF alone (HD‐NK+AMF), incubated with IONCs P1 at 37°C (HD‐NK+IONCs P1), and treated with IONCs P1 and MHT (HD‐NK+IONCs P1+MHT).

Following the treatment, cells were washed to remove excess MNPs, resuspended in cytokine‐free medium, and placed on ice to preserve their post‐treatment state, reflecting peak stress levels for functional assessment just after the mild‐MHT. Next, NK cells were stained with monoclonal antibodies and analysed via flow cytometry (Figure 6A–E). Notably, the expression level of most of the surface receptors examined was not affected by mild‐MHT treatment. Only CD16 and DNAM‐1 showed a statistically significant reduction of their expression following IONCs exposure, regardless of MHT application. Remarkably, they remained clearly detectable on the cell surface and, especially for CD16, for which a more pronounced effect was observed, with MFI levels that remain functionally relevant (Figure S7).

Both receptors are known substrates of ADAM family proteases, particularly ADAM17, whose activation may be enhanced by nanoparticle uptake and hyperthermia‐induced stress. This modulation likely reflects receptor shedding or endocytosis rather than a detrimental effect. Indeed, increased ADAM activity could promote extracellular matrix remodelling and facilitate NK cell infiltration into tumor tissues, suggesting a potentially advantageous adaptive response [55].

Similarly, a slight decrease was also observed in the level of expression of CD69, even if not statistically meaningful, thus indicating that NK cells still preserve their activation status. Given the importance of surface receptors in NK cell activation and anti‐tumor activity, we next assessed the degranulation capacity of treated HD‐NK cells by measuring CD107a expression in response to neuroblastoma (SH‐SY5Y) and glioblastoma (U87) targets (Figure 6E,F). None of the conditions impaired degranulation: IONCs P1+MHT‐treated cells exhibited strong degranulation responses (31% for SH‐SY5Y, 41% for U87), comparable to controls (Figure 6F). Notably, despite the observed decrease in DNAM‐I and CD16 expression, overall receptor profiles and degranulation activity remained intact or even enhanced.

Overall, these results confirm that IONCs internalization and mild‐MHT preserve HD‐NK cell functionality, including cytotoxic response and surface receptor expression critical for tumor recognition and migration. To further demonstrate the versatility of the mild‐MHT protocol, we extended its application to T cells, given their central role in ACT and the relevance of NP‐based tracking for immune monitoring in therapeutic settings. As an initial validation, we tested Jurkat T cells incubated with IONCs P2, the same MNPs previously tested on NK‐92 cells. Post‐treatment analysis confirmed that cell viability of T cells was unaffected by AMF exposure (Figure 5D) and nanoparticles were successfully taken up (Figure 5E,F), with significantly higher iron levels in mild–MHT–treated T cells (∼6 pg Fe/cell) compared to those incubated at 37°C without AMF (∼2.5 pg Fe/cell). These findings demonstrate the broad applicability of the mild‐MHT protocol across different immune cell types and highlight its potential utility in imaging‐based modalities, enabling real‐time monitoring of T cell dynamics in therapeutic settings.

## 2 Conclusions

The novelty of our approach lies in the use of MHT not for its classical therapeutic heating treatment of tumors, but as a new technological tool to transiently affect immune cell membranes, enabling enhanced nanoparticle uptake within a short time. Our work introduces indeed a simple, rapid, and broadly applicable method to load immune cells with MNPs by exploiting the mild temperature increase (38–41°C) generated during MHT treatment by MNPs when exposed to a time‐varying field for a short time (1 h), without affecting cell viability or functionality (Figure 3). Indeed, the mild‐MHT protocol with the heat generated directly by MNPs under AMF induces a more efficient endocytosis of nanoparticles in cells in suspension, resulting in a higher amount of internalized iron compared to immune cells kept at the same temperature (41°C) in a thermomixer (Figure S6). We demonstrate the effectiveness of our protocol across various MNP coatings and compositions, and we validated the protocol on immune cell lines such as NK‐92 and Jurkat T cells, as well as on HD‐NK cells (Figure 5). Despite their smaller size and more delicate handling requirements, even the HD‐NK cells showed robust and quantitative MNP internalization while maintaining viability, receptor expression, and degranulation activity against glioblastoma and neuroblastoma targets. The MNPs, once associated with immune cells, are also stable and do not change over a period of 24 h as verified by elemental analysis (Figure S5). Importantly, the internalized MNPs enabled detection via MPI, supporting the potential for MPI imaging and quantification in immune cells in ACT (Figure 3).

Allogeneic (HD) or patient‐derived NK cells and γδ T cells are already used in the clinic to boost immune responses in cancer patients, produced under Good Manufacturing Practice conditions in certified facilities, ensuring regulatory compliance for patient infusion. Our mild‐MHT protocol can be easily integrated into these adoptive cell therapy workflows. Notably, the present study is mostly based on iron oxide MNPs, which are approved by the FDA as MRI contrast agents and are already used clinically in iron supplements such as Feraheme, Venofer, and Ferinject for anemia treatment. In the future, provided that MNPs can also generate heat under near‐infrared laser irradiation, we may pursue this combination as a strategy to induce heating while enhancing MNP uptake by immune cells and compare cellular uptake response when using a different source of heat rather than AMF. In summary, this simple test tube‐based approach is robust, adaptable to various immune cell types and formulations of MNPs, and holds strong potential for clinical translation, and, although obvious, no one has ever exploited the mild‐MHT principle for cell uptake. This approach opens new avenues for magnetically labelling of other cell types (i.e., stem cells, probiotics, etc.) and for using MNP‐loaded NK cells and T cells as “Trojan horses” to deliver therapeutic MNPs to tumors for combined immunotherapy and MHT, while enabling real‐time monitoring of cell biodistribution.

## 3 Materials and Methods

### 3.1 Chemicals

Poly maleic anhydride‐*alt*‐1‐octadecene (PMAO), sucrose, agarose, Dulbecco's modified Eagle's medium (DMEM, high glucose), RMPI‐1640 medium, Dulbecco's phosphate‐buffered saline (PBS), L‐glutamine solution, penicillin/streptomycin solution, fetal bovine serum (FBS), fetal calf serum (FCS), and 0.25% trypsin‐EDTA solution were obtained from Sigma–Aldrich. Human IL‐2 recombinant protein (IL‐2) was purchased from Gibco. Fixable viability stain 450 assay was purchased from BD Biosciences (BD Horizon Fixable Viability Stain 450). Ficoll‒Paque density gradient was purchased from Euroclone S.P.A. NK cell isolation kit was purchased from RosetteSep kit, StemCell Biotechnologies. Milli‐Q water filtered through 0.22 µm pore size hydrophilic filters (18.2 MΩ cm) was supplied by a Milli‐Q Integral Water Purification System. NK‐92 MI and U‐87 MG were purchased from ATCC. All cubic nanoparticles were prepared following previously reported protocols [40, 42].

### 3.2 Synthesis of MNPs and Water Transfer With P1 Polymer

IONCs were prepared according to our previous works either by thermal decomposition or by solvothermal methods,^40,42,^ while for the synthesis of Zn‐Fe NPs, we refer to the thermal decomposition protocol reported by us [40, 42, 56]. The synthesized MNPs in chloroform were transferred into water, using two main coatings. For the first type, P1, a two‐step approach is used. For the first step, poly (maleic anhydride‐alt‐1‐octadecene) (PMAO) is used to enwrap the IONCs, following a protocol developed by our group with suitable modifications [57]. 1 mL Chloroform solution of a 0.2 µM in Fe of chloroform solution of 16/18 nm‐IONCs/Zn‐Fe NPs was diluted with 70 mL of chloroform and sonicated for 5 min. In a 50‐ml single‐neck flask, a certain amount of PMAO polymer in chloroform at 137 mM was added to the MNPs solution such that the ratio of polymer monomer units per nm^2^ of the MNPs surface corresponded to 750. The solvent was evaporated using a Rotavapor (140 rpm, 50°C) under reduced pressure following the three‐step scheme: 800 mbar for 30 min, 700 mbar for 30 min, and finally 600 mbar until complete solvent evaporation. To the dried sample, 20 mL of borate buffer (pH 9) was added and sonicated at 65°C until complete solubilization of the materials (the solution appeared brownish in color with no residual color on the flask wall). The water‐soluble MNPs were concentrated by an Amicon filter tube (50 mL volume and 100 kDa in molecular weight cut off, MWCO). The sample volume was reduced to nearly 3 mL through successive centrifugation (1400 rpm for 10 min). Next, the sample was loaded on top of a sucrose gradient (2 mL of 20%, 3 mL of 40%, and 3 mL of 60% in a 14 mL ultra‐clear centrifuge tube, from top to bottom) and centrifuged at  000 rpm for 45 min. The excess of polymer, which was visible under a UV lamp, was stratified at the top of the 20% sucrose gradient. It was removed by collecting it with a syringe. MNPs, instead, were located in the 40%–60% fraction of the sucrose gradient. They were collected with a syringe and further washed with Milli‐Q water three times using an Amicon filter tube (50 mL volume and 100 kDa MWCO) to remove the excess of sucrose present in the sample. Finally, the sample was concentrated to 1 mL of final solution. In a second step, di amine Poly(ethylene glycol) (NH2–PEG–NH2, MW 2000) was attached to the polymer‐coated MNPs following a procedure developed in our group, with minor modifications [44]. 1 mL of water‐dispersed MNPs at the concentration of 0.1µM (in NPs concentration) was mixed with the same volume of a milli‐Q water solution, 20 mM of 1‐ethyl‐3‐(3‐dimethylaminopropyl)carbodiimide hydrochloride (EDC), and another 1 mL of milliQ water solution, 0.1 mM of NH_2_–PEG–NH_2_. The mixture was vigorously shaken for 1 min, and then it was placed under gentle shaking (150 rpm) for 2 h at RT. Afterward, the solution was diluted 3 times in aqueous volume to quench the reaction and washed in pure water using centrifugal filters. Finally, the sample was concentrated up to a tiny volume (∼500 µL).

### 3.3 Water Transfer With P2 Polymer

For comparing the polymer shell in terms of uptaking, we have also water transferred our IONCs with another polymer named dopamine–PIMA–amino PEG methoxy (2000) (DOPA‐PIMA PEG), and here referred to as Polymer 2, following an already published protocol with minor modifications [58]. For this purpose, first, we have synthesized an amphiphilic polymer with the backbone composed of poly(isobutylene‐alt‐maleic anhydride) (PIMA), partially functionalized with multi‐dopamine molecules and with multiple amino‐PEG‐methoxy (2000 Da) ligands [58]. The polymer was synthesized under an inert atmosphere by combining an equimolar amount of activated dopamine with triethylamine and amino‐PEG in the PIMA chain, maintaining a Dopamine:PEG:PIMA ratio of 1:1:2. The dopamine catechol group offers strong binding with the IONPs surface, while the amino‐PEG allows further extension of chemistry on the hydrophilic side. The solvent used was anhydrous dimethylformamide (DMF), and the mixture was stirred overnight at 70°C under continuous nitrogen flow. Theoretically, we consider that dopamine and PEG will occupy 100% of the possible maleic anhydride rings (39 rings in a 6000 g/mol PIMA) [43, 59]. After the reaction, the polymer converts to a thick gel solution and is dissolved in chloroform for later use. Considering the initial concentration of the MNPs in chloroform, we then calculated the amount of polymer in the same solvent to be added to the MNP solution such that it corresponds to 7 polymer molecules per nm^2^ which do correspond to 136.5 dopamine units per nm^2^. The MNPs solution and the polymer were left for 16 h under a vortexer at 1100 rpm at RT. After the ligand exchange, the particles were transferred to water using a CHCl_3_/Hexane/H_2_O liquid‐liquid extraction system. The bottom layer contained the chloroform mixture, the middle layer contained three times the volume of water, and the top layer contained three times the volume of hexane relative to chloroform (1:3:3 for a total volume of 42 mL). The mixture was vigorously shaken and left to separate. The water fraction containing the MNPs was collected from the bottom, while the upper hexane/chloroform mixture was discarded. The collected MNPs in water were filtered (0.2 µm syringe filters) and concentrated by centrifugation using Amicon filter tubes (50 mL, 100 kDa of MWCO) at 1500 rpm for 10 min, with the addition of Milli‐Q water during the first two cycles.

### 3.4 Immune Cell Lines

#### 3.4.1 NK Cells

NK‐92 MI human NK cell line, referred to in the whole paper as NK‐92 cells, was purchased from ATCC, and they were cultured in RMPI 1640 medium, 10% FBS, 1% penicillin/streptomycin, 1% L‐glutamine, and 600 U/mL of IL‐2 at 37°C, 5% CO2, and 95% relative humidity according to the manufacturer's ATCC protocols. Cells were split according to the ATCC manufacturer's protocol and used at passage numbers between P10 and P15.

#### 3.4.2 Jurkat Cells

Jurkat T cells, purchased from ATCC, were cultured in RMPI 1640 medium, 10% FBS, 1% penicillin/streptomycin, 1% L‐glutamine at 37°C, 5% CO2, and 95% relative humidity. Cells were split according to the ATCC manufacturer's protocol and used at passage numbers between P10 and P15.

### 3.5 NK cells from HDs

Mononuclear leukocytes were isolated by Ficoll‒Paque density gradient from the peripheral blood of HDs admitted at the blood transfusion centre of IRCCS Ospedale Policlinico S. Martino after obtaining informed consent (Study approved by the Ligurian Ethics Committee, 39/2012, number CER Liguria DB id 10125). NK cells were purified using an NK cell isolation kit. After isolation, NK cells were cultured in round‐bottom 96‐well plates in 200 µL of RPMI 1640 medium supplemented with 10% FBS, 1% penicillin/streptomycin, 1% L‐glutamine, and 600 U/mL of IL‐2 and containing a feeder layer made of irradiated peripheral blood mononuclear cells (3*10^6^ cells) and the 721.221 (1*10^6^) lymphoblastoid cell line. After 3 weeks, the expanded polyclonal NK cell population was checked for purity by one‐color flow‐cytometry analysis (FACSCalibur Becton Dickinson & Co, Mountain View, CA) using monoclonal antibodies specific for NK cells (i.e., NKp46) or other PBMCs' markers. Fluorochrome‐labelled secondary reagent only or isotype‐matched antibodies were used as negative controls. The analysis was performed according to the flow cytometric standard procedure. Briefly, 1*10^5^ cells were incubated with 50 µL of the primary monoclonal antibodies (mAb) and incubated for 30 min at 4°C. Cells were then washed once in PBS to remove the excess antibody and then incubated with the secondary mAb for 30 min at 4°C. After this incubation, the residual secondary reagent was removed by washing, making the cells ready to be analysed. In every experimental session, the flow‐cytometer performances were controlled, and the reproducibility of the fluorescence intensity was aligned using calibrated microspheres (Becton Dickinson & Co, Mountain View, CA).

### 3.6 Tumor Cells

U87 MG human glioblastoma cells (ATCC HTB‐14) were cultured in T75 flasks as a monolayer in DMEM high‐glucose supplemented with 10% heat inactivated FBS, 2% penicillin−streptamycin (10000 U/mL), and 1% L‐glutamine (200 mM) at 37°C, 5% CO2, and 95% relative humidity. All of the cell culture reagents were purchased from Gibco. SH‐SY5Y neuroblastoma cell line, kindly provided by Dr. F. Pastorino (IRCC istituto Giannina Gaslini, Genoa, Italy), was cultured in the presence of RPMI 1640 medium supplemented with 2 mM glutamine, 50 mg/mL penicillin, 50 mg/mL streptomycin, and 10% heat‐inactivated FCS. Cells were split when they reached 80% confluency and used at passage numbers between P10 and P15. All reagents were purchased from Sigma‐Aldrich.

### 3.7 MHT on NK Cells

1.2*10^6^ NK92 cells or HD‐NK cells were mixed with either UV‐sterilized (1 h) IONCs P1 or IONC P2 or with Zn Fe P1 NPs at a concentration of 3 g Fe/L. Then, the cell samples were exposed to 2 cycles of 30 min each of mild‐MHT, at fixed temperature (41°C) and fixed frequency (182 kHz) with a field intensity varying in the range 17–24 kA/m. After MHT, cell pellets were recovered and washed by centrifugation (1400 rpm for 7 min) with two cycles of centrifugation and media resuspension steps to remove the excess of MNPs not associated with the cells.

### 3.8 Iron Quantification by Means of Elemental Analysis

For the iron quantification, approximately 1*10^6^ NK cells were washed to remove the free MNPs (with or without MHT exposure) and pellet at 1400 rpm for 7 min. The supernatant was discarded, and to the cell pellet, 2 mL of concentrated H_2_O_2_/HNO_3_ solution (1:1 volume ratio) was added and kept in a water bath at 65°C for 2 h under sonication for material digestion. Analysis was also conducted on cells not treated with MNPs. The solution was diluted to 10 mL with Milli‐Q water and filtered with an RC filter (0.45 µm) before Fe quantification using ICP‐OES. Three iron measurements were made for each sample. For the 24 h iron measurements, cells were replated under standard culture conditions immediately after 1 h MHT. After 24 h, the cells were collected, the materials digested as described above, and the iron content was quantified to determine the amount per cell.

### 3.9 TEM Imaging of NK Cells

After the incubation of NK cells with MNPs in the absence or in the presence of MHT, 1*10^6^ cells were washed twice by centrifugation and then pellet at 1400 rpm for 7 min. After that, they were fixed with a solution of 2% (in volume) of Glutaraldehyde in medium and incubated for 45 min at RT. Then, the suspension was centrifuged at a speed of 5000 rpm to obtain a solid pellet. Next, 2% Glutaraldehyde in 0.1 M Na‐Cacodylate Buffer pH 7.4 was gently dropped in the well to avoid pellet disaggregation and incubated for 1 h at RT. After this incubation, the sample was washed three times with 0.1 M Sodium Cacodylate, fixed, and processed with 1% Osmium Tetroxide solution and dehydrated with ethanol (from 70% to 100%). The pellet was then embedded into resin and then sliced for TEM imaging at about 100 nm with an ultramicrotome (Leica Ultracut EM UC6 – Cryo‐ultramicrotome).

### 3.10 Cell Viability

Cell viability was assessed by flow cytometry using the fixable viability stain 450 assay. After 24 h post‐MHT, 2*10^5^ immune cells/well were collected and washed once in sodium azide‐ and protein‐free Dulbecco's Phosphate Buffered Saline (1X DPBS). Then, they were resuspended in 100 µL of a solution containing the death FVS450 dye in PBS (1:1000 concentration) and incubated for 30 min at 4°C. After the incubation, cells were washed once with FACS Buffer (2% FBS in PBS) and then resuspended in the same buffer before their analysis (FACSCalibur Becton Dickinson & Co, Mountain View, CA).

### 3.11 Transwell Migration Assay

The migratory capacity of NK‐92 cells was evaluated using a Transwell‐based chemotaxis assay. Briefly, 600 µL of U87‐conditioned medium (collected after 24 h of culture exposure) was added to the lower compartments of 24‐well Transwell plates (3.0 µm pore size, Corning). NK‐92 cells (1*10^5^) were resuspended in 100 µL of serum‐free medium and seeded into the upper inserts of the transwell. Following an 18 h incubation period, the migrated cells were recovered from the bottom chambers by centrifugation (300 rcf for 5 min at 4°C). The resulting cell pellet was resuspended in 100 µL of PBS, and viable cells were quantified using a hemocytometer through Trypan blue exclusion staining. Three cell groups were considered: untreated NK‐92 cells, NK‐92 cells incubated with IONCs P1 at 37°C for 1 h, and NK‐92 cells incubated with IONCs P1 and exposed to two cycles of mild‐MHT (fixed frequency 182 kHz, targeted temperature reached of 41°C).

### 3.12 NK Cell Degranulation Assay

NK cell degranulation was assessed by flow cytometry on NK92 or HD‐NK cells co‐cultured with U87 or SH‐SY5Y target cells. Briefly, 1*10^6^ NK cells were mixed with IONCs P1 at 37°C or exposed to 2 cycles of MHT as previously described. NK cells not exposed to any treatment in the absence of MNPs were used as a control. After their incubation with IONC P1, cells were collected, washed of the excess of MNPs, suspended in complete RPMI medium, and co‐cultured with either U87 or SH‐SY5Y in a 96‐well plate at an effector/target (E:T) ratio of 1:1 (2*10^4^ NK cells: 2*10^4^ tumor cells). A eFluor660‐conjugated mAb specific for the CD107a molecule was added to the co‐culture. Then the plate was centrifuged at 1000 rpm for 5 min and incubated for 3 h at 37°C. Cells were collected, washed twice with FACS Buffer, and then analysed by flow cytometry (FACSCalibur Becton Dickinson & Co, Mountain View, CA).

### 3.13 NK Cell Phenotype

This analysis was performed by flow cytometry using the following mAbs available in the Molecular Immunology Lab at the University of Genoa (Genoa, Italy): AZ20 (IgG1, anti‐NKp30), BAB281 (IgG1, anti‐NKp46), ON72 (IgG1, anti‐NKG2D), KRA236 (IgG1, anti‐DNAM‐I), C227 (IgG1, anti‐CD69), C127 (IgG1, anti‐CD16), MA152 (IgG1, anti‐NKp80), DF200 (IgG1, anti‐KIRs), Z270 (IgG1, anti‐NKG2A). Anti‐CXCR3 (IgG1) and anti‐CXCR4 (IgG2b) mAbs were purchased from R&D Systems (Minneapolis, MN).

### 3.14 MPI Analysis

MPI analyses were conducted using the Momentum CT device. Calibration lines were produced to determine the relationship between iron content in IONCs P2 and the MPI signal. To construct this line, serial dilutions of IONCs P2 were prepared in a final volume of 10 µL. The following iron contents were tested: 20, 15, 11, 7, 3.5, 1.7, 0.8, and 0.4 µg. Samples were located in a sample holder, and then 2D multi‐projection images and relaxometer analysis were conducted for each sample. To quantify the MPI signal of the samples, a circular region of interest (ROI) with a fixed radius was manually drawn over the known sample positions. For calibration, the Hounsfield Unit (HU) values of the pixels within each ROI were summarized and correlated with the total iron content, as determined by ICP‐OES analysis. A linear correlation with a correlation coefficient, R^2^ value of 0.9900, was obtained, consistent with theoretical expectations. For the cellular samples, after the mild‐MHT protocol, 1*10^6^ cells were washed to remove excess NPs, then transferred into a 200 µL Eppendorf tube in a 10 µL final volume and imaged as the calibration samples. The total MPI signal was plotted against iron content to derive calibration lines, and this relationship was used to quantify iron content in cell pellets.

### 3.15 Statistical Analysis

Statistical analysis was performed on data from at least three biologically independent experiments or biological replicates, as indicated in the figure legends. Normal data distribution was assumed. Results are presented as the mean ± sem. Statistical significance between two groups was assessed using unpaired Student's *t*‐test (two‐sided). For comparisons involving more than two groups, statistical significance was determined using one‐ or two‐way ANOVA, followed by Tukey's or Sidak's post hoc multiple comparisons test. Statistical significance was defined as *p* < 0.05, ^*^ *p* < 0.01, ^**^ *p* < 0.001, and ^***^ *p* < 0.0001. Exact *p*‐values are reported in the figure legends where applicable. All statistical analyses were conducted using GraphPad Prism 9.

## Conflicts of Interest

Some of the authors disclose the submission of one patent associated with this research (Ref. IIT PT240732). They report no other financial conflicts of interest.

## Supporting information




**Supporting File**: smtd70712‐sup‐0001‐SuppMat.docx.

## Data Availability

Extra data is included in the ESI. If needed, more detailed information or images, the authors will provide them upon request via email.
