# Supplementary material for: Functional Repurposing of Magnetic Nanoparticle‐Assisted Hyperthermia for Immune Cell Labelling and Tracking
Source: Small Methods. 2026 Jun 11;10(18):e02416. doi: 10.1002/smtd.202502416 (PMC13599683; doi:10.1002/smtd.202502416)
Supplement: Supplementary file 1 [file SMTD-10-e02416-s001.docx]

Supporting information

Functional Repurposing of Magnetic Nanoparticle-Assisted Hyperthermia for Immune Cell Labelling and Tracking

*Giulia E.P. Nucci^1^, Chiara Vitale^2^, Alessandro di Girolamo^1^, Niccolò Silvestri^1^, Stefano Persano^1^, Lorenci Gjurgjaj^1^,^3^, Cristina Bottino^2,4^, Roberta Castriconi^2^*, Teresa Pellegrino*^1^*

^1^ Italian Institute of Technology, via Morego 30 16163, Genoa

^2^ Department of Experimental Medicine (DIMES), University of Genoa, 16132 Genoa, Italy

^3^ The Open University Affiliated Research Center, Italian Institute of Technology, via Morego 30, Genoa, 16163, Italy

^4^ Laboratory of Clinical and Experimental Immunology, IRCCS Istituto Giannina Gaslini, 16147 Genova, Italy

*[teresa.pellegrino@iit.it](mailto:*teresa.pellegrino@iit.it); [roberta.castriconi@unige.it](mailto:roberta.castriconi@unige.it)

**Table S1: Table summarizing the principal colloidal properties of each nanoparticle used in the MHT protocol.**

| **Samples** | **TEMCore Size** | **Z-avg**  **(nm)** | **PDI** | **D_H_ by intensity (nm)** | **D_H_ by volume (nm)** | **D_H_ by number (nm)** | **Z-potential (meV)** |
| --- | --- | --- | --- | --- | --- | --- | --- |
| **IONCs P1** | 15$\pm$1 | 97 | 0.229 | 121$\pm$25 | 70$\pm$48 | 41$\pm$16 | -28$\pm$7 |
| **IONCs P2** | 15$\pm$1 | 97 | 0.206 | 125$\pm$56 | 91$\pm$55 | 58$\pm$22 | -35$\pm$7 |
| **Zn-Fe P1** | 18$\pm$2 | 70 | 0.186 | 86$\pm$40 | 53$\pm$26 | 37$\pm$12 | -28$\pm$9 |

**Core size**: trasmission electron microscopy (TEM) size dimension of the as-synthesized MNPs (nm)

**Z-avg**: weighted avarage of hydrodynamic diameter from DLS (nm)

**PDI**: polydispersity index indicating size distribution width; lower values reflect more monodisperse samples.

**Dh:** hydrodynamic diameter by intensity, volume or number from dynamic light scattering (DLS) with full width at half maximum (FWHM) in nm

**Z-potential:** surface charge ( mV)

**Fig. S1: Optical images of NK-92 taken 24h after exposure to different thermal treatment conditions when using the thermomixer as a heating source.** The morphology of the cells at 41 °C for 60 or 90 minutes looks very similar to that at 37°C. In contrast, at 43 °C, progressive morphological changes became evident, and with prolonged thermal treatment, from 60 to 90 minutes, the cells increasingly lose their ability to form clusters, indicated by the disappearance of black dots. The magnification used is 10X.

***Fig. S2: Evolution of the magnetic field amplitude (H) for each MNPs used during mild-MHT treatments****. The frequency was fixed at 182 kHz and the temperature at 42 °C. The field was initially increased to high values (40–44 kA/m) to rapidly reach the target temperature, and then reduced and stabilized (14–24 kA/m) to maintain it.*

**Fig. S3: Evolution of the magnetic field amplitude (H) and the temperature during mild-MHT treatments**. (A) Representative profiles of the applied magnetic field amplitude over 60 minutes of treatment at a fixed frequency (f = 182 kHz) and at a fixed temperature (T=41°C). (B) Representative temperature profiles recorded for a solution of IONCs P1 alone and IONCs P1 in presence of cells. No significant adjustments in the magnetic field amplitude were required during the 1-h AMF protocol to maintain the target temperature for the cells sample in presence of IONCs.

**Fig. S4 :** Temperature profile and Viability of T cells only after exposure to an AMF and without nanoparticles addition. A) Temperature profile (in blue) for T cells only exposed at 182kHz frequency and magnetic field intensity profile of 40 kA/m (in grey) for 1 hour and B) corresponding cell viability of T cells exposed at those conditions versus untreated control T cells kept at 37°C. Cell viability, tested by Presto Blue, stay up to 90 % indicating any toxic effect of the AMF on the immune cells. For each data three independent experiments were carried out.

**Fig. S5: MPI signal calibration.** (A) Schematic representation of the holder used for MPI imaging with the relative disposition of the samples used for the calibration. (B) Relaxometer data showing the point spread functions for all the samples used in the calibration.

***Fig. S6: Evaluation of iron uptake overtime.*** *Elemental analysis evaluation of iron on HD-NK immediately after the 1-hour mild-MHT protocol (no Delay) and after a delay time of 24h (24h Delay). For each data three independent experiments were carried out.*

**Fig. S7: Representative flow cytometry analysis** **of HD-NK after incubation with IONCs P1 and MHT.** The panels show the expression of the indicated surface markers. The analysis was performed in untreated control cells (HD-NK), HD-NK+AMF, HD-NK+IONCs P1, and HD-NK+IONCs P1+MHT.
